# Evidence for a role of NTS2 receptors in the modulation of tonic pain sensitivity

**DOI:** 10.1186/1744-8069-5-38

**Published:** 2009-07-06

**Authors:** Geneviève Roussy, Marc-André Dansereau, Stéphanie Baudisson, Faouzi Ezzoubaa, Karine Belleville, Nicolas Beaudet, Jean Martinez, Elliott Richelson, Philippe Sarret

**Affiliations:** 1 Department of Physiology and Biophysics, Faculty of Medicine and Health Sciences, Université de Sherbrooke, Sherbrooke, Quebec, Canada; 2 Department of Psychiatry and Psychology, Mayo Clinic College of Medicine, Jacksonville, FL 32224, USA; 3 CNRS UMR 5247, IBMM, Universités Montpellier 1 & 2, 34095 Montpellier, France

## Abstract

**Background:**

Central neurotensin (NT) administration results in a naloxone-insensitive antinociceptive response in animal models of acute and persistent pain. Both NTS1 and NTS2 receptors were shown to be required for different aspects of NT-induced analgesia. We recently demonstrated that NTS2 receptors were extensively associated with ascending nociceptive pathways, both at the level of the dorsal root ganglia and of the spinal dorsal horn. Then, we found that spinally administered NTS2-selective agonists induced dose-dependent antinociceptive responses in the acute tail-flick test. In the present study, we therefore investigated whether activation of spinal NTS2 receptors suppressed the persistent inflammatory pain symptoms observed after intraplantar injection of formalin.

**Results:**

We first demonstrated that spinally administered NT and NT69L agonists, which bind to both NTS1 and NTS2 receptors, significantly reduced pain-evoked responses during the inflammatory phase of the formalin test. Accordingly, pretreatment with the NTS2-selective analogs JMV-431 and levocabastine was effective in inhibiting the aversive behaviors induced by formalin. With resolution at the single-cell level, we also found that activation of spinal NTS2 receptors reduced formalin-induced *c-fos *expression in dorsal horn neurons. However, our results also suggest that NTS2-selective agonists and NTS1/NTS2 mixed compounds differently modulated the early (21–39 min) and late (40–60 min) tonic phase 2 and recruited endogenous pain inhibitory mechanisms integrated at different levels of the central nervous system. Indeed, while non-selective drugs suppressed pain-related behaviors activity in both part of phase 2, intrathecal injection of NTS2-selective agonists was only efficient in reducing pain during the late phase 2. Furthermore, assessment of the stereotypic pain behaviors of lifting, shaking, licking and biting to formalin also revealed that unlike non-discriminative NTS1/NTS2 analogs reversing all nociceptive endpoint behaviors, pure NTS2 agonists specifically inhibited paw lifting, supporting a role of NTS2 in spinal modulation of persistent nociception.

**Conclusion:**

The present study provides the first demonstration that activation of NTS2 receptors produces analgesia in the persistent inflammatory pain model of formalin. The dichotomy between these two classes of compounds also indicates that both NTS1 and NTS2 receptors are involved in tonic pain inhibition and implies that these two NT receptors modulate the pain-induced behavioral responses by acting on distinct spinal and/or supraspinal neural circuits. In conclusion, development of NT agonists targeting both NTS1 and NTS2 receptors could be useful for chronic pain management.

## Background

Endogenous neurotensin (NT) as well as centrally administered NT analogs produce dose-dependent analgesic effects in either somatic or visceral pain paradigms [1]. Site-specific microinjections of NT also result in a mu-opioid receptor-independent antinociceptive response in the hot-plate and acetic acid-induced writhing tests in rodents [2-6]. Molecular cloning and pharmacological data have demonstrated the existence of at least three subtypes of NT receptors referred to as NTS1, NTS2, and NTS3 [7-9]. However, there is now evidence that the analgesic action of NT is mediated through activation of both G-protein coupled receptors, NTS1 and NTS2 [1,10-12].

Originally, several studies pointed to the levocabastine-sensitive low-affinity NTS2 subtype as a major NT antinociceptor. The earliest indication was that the analgesic effectiveness of a panel of metabolically stable peptide and pseudopeptide analogs of NT did not correlate with their binding affinities for NTS1 [13,14]. Among them, intracerebroventricular (i.c.v.) or intrathecal (i.t.) injection of the NTS2-selective agonist, JMV-431, induced strong antinociceptive responses in acute and visceral pain tests in rodents [13,15]. Similarly, i.t. or intra-rostroventromedial medulla (RVM) administration of both selective NTS2 analogs, levocabastine and β-lactotensin engendered analgesia in rats, as measured in the tail-flick test [15-19]. Furthermore, i.c.v. administration of the NT antagonist SR142948A, which recognizes both high- and low-affinity NT sites, blocked NT-induced analgesia, whereas the relative selective NTS1 antagonist SR48692 did not [14,20-22]. The contribution of NTS2 in regulating nociceptive processes was further demonstrated by knockdown of the target protein by antisense approaches. Indeed, sustained delivery of antisense oligodeoxynucleotides (ODN) directed against NTS2 to the brain, or spinal administration of selective NTS2 dicer-substrate small interfering RNA (siRNA), markedly reduced NT-induced antinociception [13,19,23]. Accordingly, the generation of NTS2-deficient mice confirmed the involvement of this receptor in pain processing [24]. NTS2 receptors are also well positioned to modulate nociception at several different levels. Thus, *in situ *hybridization, autoradiographic binding, and immunohistochemical studies have revealed the presence of NTS2 receptors in cerebral regions implicated in the descending control of nociceptive inputs such as the periaqueductal gray (PAG), nuclei raphe magnus, dorsalis, pallidus, and gigantocellular pars alpha [25-32]. Consistent with a role for NTS2 receptors in the mediation of NT's spinal antinociceptive actions, high levels of NTS2 immunolabeling were observed within dorsal root ganglion cells and their central afferent terminals in the superficial laminae of the dorsal horn, as well as in postsynaptic elements throughout the dorsal horn of the rat spinal cord [15].

NT also exerts its central analgesic effects by acting at NTS1 sites. Intra-RVM injection of the selective-NTS1 receptor agonist PD149163 produces dose-dependent thermal antinociception that is blocked by the NTS1 antagonist SR48692 [16,17]. Receptor knockout and knockdown strategies support the pharmacological data. Mice deficient in or lacking the NTS1 receptor fail to exhibit NT-induced antinociception to thermal stimuli [33,34]. The presence of high levels of NTS1 mRNA and proteins in regions implicated in pain regulation such as the RVM, PAG, lumbar dorsal root ganglion (DRG) and spinal cord neurons also suggests the role of NTS1 in nociceptive modulation [16,35-39]. Accordingly, we recently demonstrated that i.t. NTS1 agonists significantly reverse the formalin-induced nociceptive behaviors, providing the first demonstration for a direct involvement of NTS1 receptors in NT-induced analgesia in a model of persistent pain [37]. There is, however, substantial evidence that receptors different from NTS1 also regulate nociceptive signaling in a formalin tonic pain model. The involvement of additional receptors is indeed suggested by the finding that the reversal by SR48692 of the antinociception produced by PD149163 was more complete than of that induced by NT69L, a peptidase resistant NT analog which binds to both NTS1 and NTS2 receptors with high affinity, but in a non-selective manner [37].

The aim of the present study was, therefore, to evaluate the potential implication of NTS2 receptors in persistent pain. To test this possibility, antinociception produced by spinal administration of selective NTS2 agonists was assessed in rats receiving intraplantar formalin into the right hind paw. Expression of the transcription factor c-*fos *is used as a functional marker to identify the spinal neurons that are activated by different forms of noxious stimulation. We then investigated whether i.t. NTS2 agonists suppressed formalin-evoked c-*fos *protein-like immunoreactivity in the rat lumbar spinal cord.

## Materials and methods

### Cell culture

The human embryonic kidney (HEK 293) cell line stably expressing either rNTS1 or rNTS2 were cultured in 175 cm^2 ^tissue culture flasks at 37°C in Dulbecco's modified Eagle's medium (DMEM) with 2 mM L-glutamine. The medium was supplemented with 5% fetal bovine serum, 0.1 mM nonessential amino acid, 1 U/ml penicillin and 1 mg/ml Geneticin (G418), in a humidified atmosphere of 95% air, 5% CO_2_. Cells were fed every 2 days with 20 ml of medium. 70–80% confluent HEK 293 cells were washed twice with phosphate-buffered saline solution, scraped into 50 mM Tris-HCl (pH 7.4) buffer containing 1 mM EDTA and 250 mM sucrose and centrifuged at 15,000 g for 12 min at 4°C. Pellets were then re-suspended in hypotonic TE buffer (50 mM Tris-HCl (pH 7.4) buffer containing 1 mM EDTA) and membrane homogenates were recovered by centrifugation for 30 min at 4°C. The final protein concentration was determined with the use of the BCA assay kit (Pierce Biotechnology Inc, Rockford, IL).

### Radioligand Binding Studies

For competition binding experiments, cell membranes (20 μg per assay for rNTS1 and 100 μg per assay for rNTS2) were incubated with 2 nM [^3^H]-NT for 45 min at 37°C in the binding buffer (50 mM Tris-HCl, pH 7.4, containing 1 mM EDTA, 40 μg/ml bacitracin, and 1% bovine serum album), in the presence of increasing concentrations (from 10^-11 ^to 10^-5^) of NT, NT69L (synthesized by E. Richelson), levocabastine (kindly provided by Janssen Research, Beerse, Belgium) and JMV-431 (synthesized by J. Martinez). Binding was terminated by two successive additions of 3 ml of ice-cold buffer and filtering under vacuum through GF/B filters presoaked in binding buffer containing 0.2% polyethyleneimine (48-well Brandel Harvester). Filters were placed in scintillation vials containing 5 ml scintillation liquid (Ecolite) and then counted in a beta counter. Specific binding was calculated as the difference between total binding (zero competing compound) and nonspecific binding (excess competing compound) and the IC_50 _values were determined from inhibition curves as the unlabeled ligand concentration inhibiting 50% of [^3^H]-NT-specific binding. Data were analyzed by LIGAND [40] and represented as geometric means, with the standard error of the geometric mean being calculated as described in [41].

### Animals, housing and habituation

Experiments were performed with adult male Sprague-Dawley rats (250–300 g; Charles River, St-Constant, QC, Canada) kept on a 12 h light/dark cycle and allowed *ad libitum *access to food and water. Animals were individually acclimatized to Plexiglas enclosures for 3 consecutive days prior to testing. The formalin test was always performed by three different experimenters in a quiet room and between 08.00 AM and 12.00 PM to reduce any variation related to circadian rhythm. The experimental procedures in this study were approved by the Animal Care Committee of the University of Sherbrooke and were in accordance with policies and directives of the Canadian Council on Animal Care.

### Behavioral studies

#### Intrathecal administration of NTS2 agonists before formalin injection

Behavioral experiments aimed at establishing the effects of NT analogs and NTS2-selective agonists on formalin-induced nocifensive behaviors. To this end, rats were lightly anesthetized with isoflurane (Abbott Laboratories, Montreal, QC, Canada) and injected intrathecally, at the L5-L6 intervertebral space, with either NT (6 μg/kg), NT69L (5 μg/kg), JMV-431 (5 to 60 μg/kg), or levocabastine (0.5 and 5 μg/kg) diluted in 30 μl of physiological saline (0.9% NaCl), 5 min before formalin administration. Control animals received physiological saline.

#### Formalin test

Antinociception was assessed using the formalin test as a model of persistent pain. For this purpose, rats were placed for a 60-min habituation period in the experimentation room. Thereafter, rats received a 50 μl subcutaneous injection of diluted 2% formaldehyde (i.e. 5% formalin, Fisher Scientific, Montreal, QC, Canada) into the plantar surface of the right hind paw.

Following this, the rats were placed in clear plastic chambers (30 × 30 × 30 cm) positioned over a mirror angled at 45°, in order to allow an unobstructed view of the paws, and their behaviors were recorded for the next 60 min. An intraplantar injection of formalin produced the biphasic nociceptive response typical of this tonic pain model [42]. The two distinct phases of spontaneous pain behaviors that occur in rats are proposed to reflect a direct effect of formalin on sensory receptors (phase 1) and a longer lasting pain due to inflammation and central sensitization (phase 2). Nocifensive behaviors were assessed using a weighed score as described previously [43,44]. A nociceptive mean score was determined for each 3 min period during the recording time by measuring the amount of time spent in each of four behavioral categories: 0, the injected paw is comparable to the contralateral paw; 1, the injected paw has little or no weight placed on it; 2, the injected paw is elevated and is not in contact with any surface; 3, the injected paw is licked, bitten, or shaken. The weighted nociceptive score, ranging from 0 to 3, is calculated by multiplying the time spent in each category by its assigned weight category, summing these products and dividing by the total time for each 3 min block of time. The total area under the curve (A.U.C.) for the inflammatory phase (phase 2) was calculated between 21 to 60 min for each animal. Phase 2 was further subdivided in early (21–39 min) and late (40–60) phases and analyzed as separate events [45]. Formalin-induced pain related behavior may also be quantified by monitoring the number and the duration of episodes of different behavioral endpoints [46]. The cumulative time spent in both lifting (state 2) and flinching/licking/biting (state 3), reflecting behavioral reactions integrated at different CNS levels, were also determined during the inflammatory phase (21 to 60 min following formalin) [42].

#### Statistical analysis

Data are presented as means ± standard errors of the mean (S.E.M.). All calculations and statistical analysis were performed using Prism 4.0 and Instat 3.05 (Graph Pad Software, San Diego, CA, USA). Nociceptive scores over the 3 min time blocks were analyzed using a two-way analysis of variance for repeated measures, with comparisons between experimental groups and the control group at each time interval using Bonferroni's *post hoc t-*test. A one-way ANOVA followed by Dunnett's *post hoc t*-test was used to determine the significance of differences in the A.U.C. and in the time spent in states 2 and 3. A difference in responses between groups was considered significant with *P *values; * *P *< 0.05 and ** *P *< 0.01.

### c-fos immunohistochemistry

The immunohistochemical study was restricted to an analysis of the effect of intrathecal pretreatment with either 30 μg/kg of JMV-431, 5 μg/kg levocabastine, or saline on the expression of c-*fos*-like immunoreactivity (c-*fos*-LI) by intraplantar injection of 5% formalin into the right hind paw. At 45 min following formalin injection, rats were deeply anesthetized and perfused intraaortically with ice-cold 4% paraformaldehyde (Fisher Scientific, Montreal, QC, Canada) in 0.1 M PB, pH 7.4. The spinal cord was isolated and then cryoprotected overnight at 4°C in 30% sucrose in phosphate-buffered saline (PBS). Prior to tissue sectioning, an incision was made in the ventral horn of the contralateral side to allow spatial positioning of the spinal cord for further analysis. Transverse frozen sections (35-μm-thick) were then cut from the lumbar segment (L4-L5) of the spinal cord with a sliding microtome and processed by a free-floating slice immunohistochemistry procedure. After elimination of endogenous peroxidase activity with 0.3% hydrogen peroxide in PBS for 1 h, the tissue sections were rinsed three times in PBS and incubated for 1 h at RT in a blocking solution of 3% normal goat serum (NGS) and 0.3% Triton X-100 in PBS. After washing, free-floating sections were incubated overnight at RT with the rabbit anti-*c-fos *polyclonal antibody diluted 1:1000 in PBS (sc-52, Santa Cruz Biotechnology, Santa Cruz, CA, USA), containing 1% NGS and 0.3% Triton X-100. The tissue was then washed three times in PBS and transferred to a goat anti-rabbit biotinylated secondary IgG complex (1:200 in 1% NGS and 0.3% Triton X-100 in PBS; Vector, Burlingame, CA, USA) for 1 h at RT followed by exposing to avidin-biotin horseradish peroxydase complex (1:100; Vectastain ABC-Elite kit, Vector Laboratories, Burlington, ON, Canada) for 1 h at RT. After the final wash with PBS, the chromagen was developed using nickel intensified 3,3'-diaminobenzidine (DAB, 0.01%) and 0.3% H_2_O_2_. Finally, the sections were thoroughly rinsed with PBS, mounted onto slides, dehydrated in a series of graded alcohols, defatted in xylene, and placed on coverslips.

### Counting of c-fos protein immunoreactive nuclei

The greatest numbers of labeled neurons evoked by formalin were detected at the L4-L5 levels, corresponding to segmental innervation of the rat plantar hind paw [47]. Sections were visually scanned and photographed using a bright-field microscope (Leica DM4000B; Leica Microsystems, Toronto, Canada). The individual sections were printed and overlaid with an acetate sheet on which the distribution of *c-fos *immunoreactive neurons was then plotted. A neuron was considered to be labeled only if the nucleus showed the characteristic staining of oxidized DAB, and was distinct from background at magnifications of 4×, 10×, and 20×. For the quantification of *c-fos *labeled neurons, each section of the spinal cord was divided into four regions of interest: the superficial laminae (laminae I, IIo and IIi), the nucleus proprius (laminae III and IV), the neck of the dorsal horn (laminae V and VI) and the deep lamina X according to the cytoarchitectonic organization of the spinal cord [48]. The number of *c-fos *immunoreactive neurons in the four defined regions was determined by averaging the counts made in 10–15 sections and expressed as mean ± S.E.M. of these values for all the rats in that treatment group for statistical analysis of the different experimental conditions. One-way analysis of variance (ANOVA) was conducted using computer software (Instat 3.05) for comparison across the experimental conditions considering the number in each defined region. A Dunnett's *t*-test was applied to assess differences between the drug and vehicle groups. A value of *P *< 0.05 was considered statistically significant.

## Results

### Competitive Binding Assays

Each NT analog was evaluated in competitive binding assays against the two cloned rat NT receptors, with binding affinities defined as the concentration of peptide required to inhibit 50% of the binding of [^3^H]-NT to either NTS1 or NTS2 (IC_50_). The binding activity data in Table 1 provide interesting information regarding the selectivity of peptidase-resistant NT agonists for NTS2 *versus *NTS1. Thus, the metabolically stable NT(8–13) analog JMV-431, protected at its N-terminus by a Boc group and by a reduced pseudopeptide bond Y(CH_2_NH) in position 11–12 binds with 250 times greater affinity to rat NTS2 (IC_50 _= 19 ± 3 nM) than to rat NTS1 (IC_50 _= 4735 ± 100 nM). The non-peptide compound levocabastine (IC_50 _= 3 ± 1 nM) was as potent as unlabeled NT (IC_50 _= 9 ± 2 nM) in inhibiting [^3^H]-NT binding to HEK293 cell membranes expressing NTS2. In addition, the NT(8–13) analog NT69L, which contains a L-*neo*-tryptophan amino acid, and exhibits high affinity for both rat (IC_50 _= 2.2 ± 0.5 nM) and human NTS1 [49] also appeared as a ligand with a good affinity for the rat NTS2 (IC_50 _= 3.7 ± 0.4 nM). Therefore, JMV-431 and levocabastine were used in the behavioral experiments as selective ligands for NTS2, whereas NT69L and NT were considered as agonists acting on both NTS1 and NTS2 in a non-discriminative manner.

**Table 1:** IC_50 _values (nM) for NTS1 and NTS2

**Compound**	**Structure**	**rNTS1**	**rNTS2**
Neurotensin	pyroGlu-Leu-Tyr-Glu-Asn-Lys-Pro-Arg-Arg-Pro-Tyr-Ile-Leu-OH	5.0 ± 0.7	9.0 ± 2
NT69L	*N-methyl*-Arg-Lys-Pro-*L-neo*-Trp-*tert*-Leu-Leu-OH	2.2 ± 0.5	3.7 ± 0.4
JMV-431	Boc-Arg-ArgPro-Tyrø(CH_2_NH)-Ile-Leu-OH	4735 ± 135	19 ± 3.2
Levocabastine	non peptidic	> 10,000	2.7 ± 1.3

Binding potencies of NT and related analogs were determined in competition experiments performed with HEK293 cells expressing either NTS1 or NTS2. Data represent means ± SEM of at least three independent experiments carried out in triplicate.

### Intrathecal administration of NTS2 agonists reduces formalin-induced persistent spontaneous nociception

Intraplantar injection of formalin into the right hind paw of saline-pretreated rats produced a typical biphasic specific nociceptive behavioral response consisting of an acute phase (0–9 min) followed by a second prolonged inflammatory phase (21–60 min) [37]. In the present experiments, we examined the contribution of NTS2 receptors to the tonic late phase of the formalin response by assessing the effects of different agonists of increasing selectivity towards NTS2. Based on previous dose-response and toxicity studies, we chose analgesic doses of NT, NT69L, JMV-431 and levocabastine that were devoid of non-specific motor effects [15,23,37].

**Figure 1:**
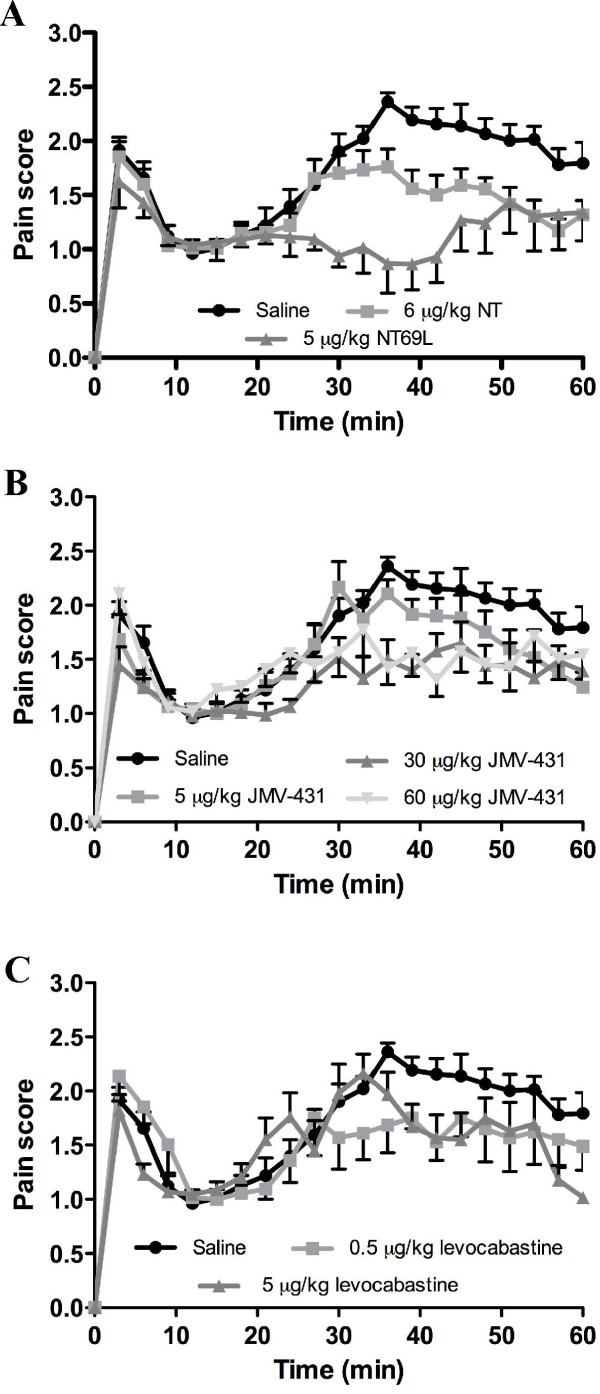
**Antinociceptive effects of acute injection of NT agonists on formalin induced pain behaviors**. **(a) **Time course of analgesic effects of NT (6 μg/kg) and NT69L (5 μg/kg). Saline and drugs were intrathecally administered 5 min before the subcutaneous injection of formalin. Nocifensive behaviors in male rats are expressed in 3 min intervals with a weighted pain score. **(b, c) **Pre-treatment with JMV-431 (5–60 μg/kg) or levocabastine (0.5–5 μg/kg) results in the inhibition of the persistent noxious chemical stimulation. Each symbol represents the mean ± SEM of determinations made in six to eight animals.

**Figure 2:**
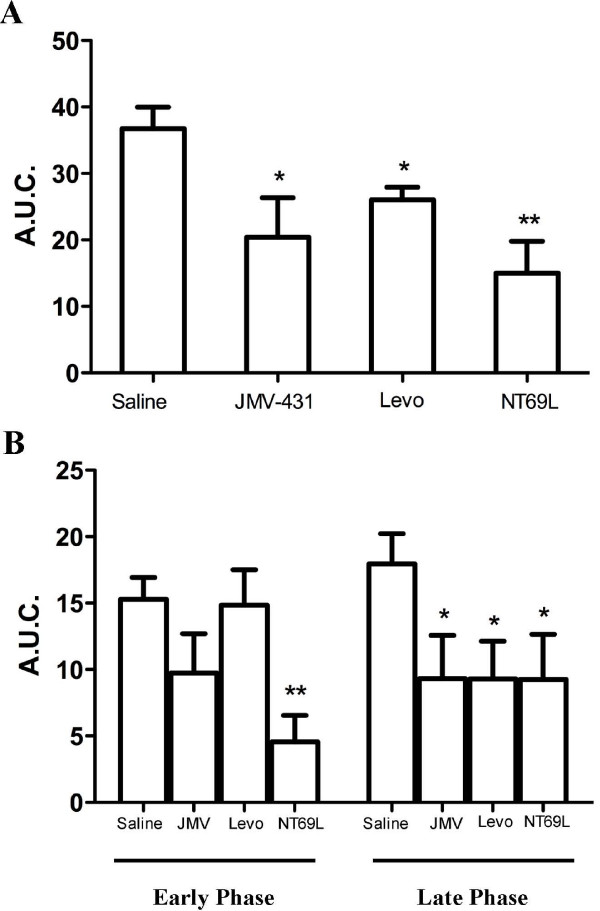
**Effects of NT compounds on the formalin-induced nociceptive behavioral responses manifested during the inflammatory phase**. **(a) **The antinociceptive response, expressed as mean area under the curve (A.U.C.), is measured during the second phase of the formalin test (21–60 min). Formalin-induced nociceptive behaviors are reduced by i.t. administration of levocabastine (5 μg/kg), JMV-431 (30 μg/kg), and NT69L (5 μg/kg) in comparison to control rats receiving saline injection. **(b) **Dissociation of phase 2 in early (21–39 min) and late (40–60 min) phases. JMV-431, levocabastine, and NT69L significantly suppressed the tonic pain response in the late phase, but only NT69L was able to induce analgesia in early phase. *P < 0.05 and ** *P *< 0.01 when compared with the vehicle-treated group (ANOVA followed by Dunnett's multiple comparison test). Data are represented as the average ± SEM.

Formalin-induced pain related behavior was first quantified by using the weighted scores method described by Dubuisson and Dennis [44]. Injected intrathecally 5 min before formalin, NT (6 μg/kg; n = 8) and NT69L (5 μg/kg; n = 8), which bind to both NTS1 and NTS2 with a similar affinity, significantly reduced pain-evoked responses during the second phase of the formalin test (Fig. 1A). Specifically, NT and NT69L pretreatment produced 48 ± 9% and 59 ± 13% reduction of pain behaviors in the tonic phase compared to vehicle-treated rats, respectively (*P *< 0.05–0.01, Fig. 2A). We then investigated whether spinally administered selective-NTS2 receptor analogs exerted antinociceptive effects following noxious chemical stimulation. As shown in Fig. 1B, injection of different doses of JMV-431 was significantly effective in inhibiting the aversive behaviors induced by formalin. All doses tested markedly attenuated the pain response to formalin during the tonic phase, producing 24 ± 11% and 45 ± 18% of inhibition at 5 μg/kg and 30 μg/kg, respectively (n = 8; *P *< 0.05–0.01, Fig. 2A). This reduction remained steady, however, despite the use of higher doses of JMV-431 (45 ± 14% of reduction in phase 2 at 60 μg/kg, n = 8; Fig. 1B). Finally, signs of nociception evoked by formalin injection were also reversed by i.t. levocabastine administration (0.5, 5 μg/kg), reaching 29 ± 5% of inhibition at the highest dose (n = 6, *P *< 0.05; Figs. 1C, 2A).

Even if all compounds tested were able to inhibit spinal nociceptive transmission induced by formalin injection, differences in the profile of the inflammatory phase were still observed between each drug. Indeed, the decrease in the pain score of phase 2 determined for both JMV-431 and levocabastine was delayed in time when compared to the NT69L profile (Fig. 1). Therefore, phase 2 was further subdivided into an early phase (21–39 min) succeeding the interphase and into a late one (40–60 min) corresponding to the sustained inflammation plateau. For comparative purposes, we evaluated the effects of equi-analgesic doses of exogenous NT agonists in terms of inhibition of phase 2 pain behaviors (Fig. 2). A statistically significant decrease in the A.U.C. was noted for the non-selective NTS1/NTS2 agonist, NT69L in both early and late phases, with a maximal decrease of 70 ± 13% and 48% ± 19% respectively (*P *< 0.05–0.01; Fig. 2B). Interestingly, the same analysis procedure applied to JMV-431 (30 μg/kg) and levocabastine (5 μg/kg) revealed that these two selective NTS2 analogs exclusively reduced the behavioral signs of formalin-induced pain by 48 ± 18% and 48 ± 16% respectively in the late phase 2, while they had no effect in the early phase (*P *< 0.05, Fig. 2B). These results suggest that NTS1 and NTS2 agonists may act differently to inhibit the nociceptive processing initiated by the persistent noxious chemical stimulation.

### Effects of intrathecal administration of NTS2 agonists on spinal and supraspinal behaviors induced by formalin

**Figure 3:**
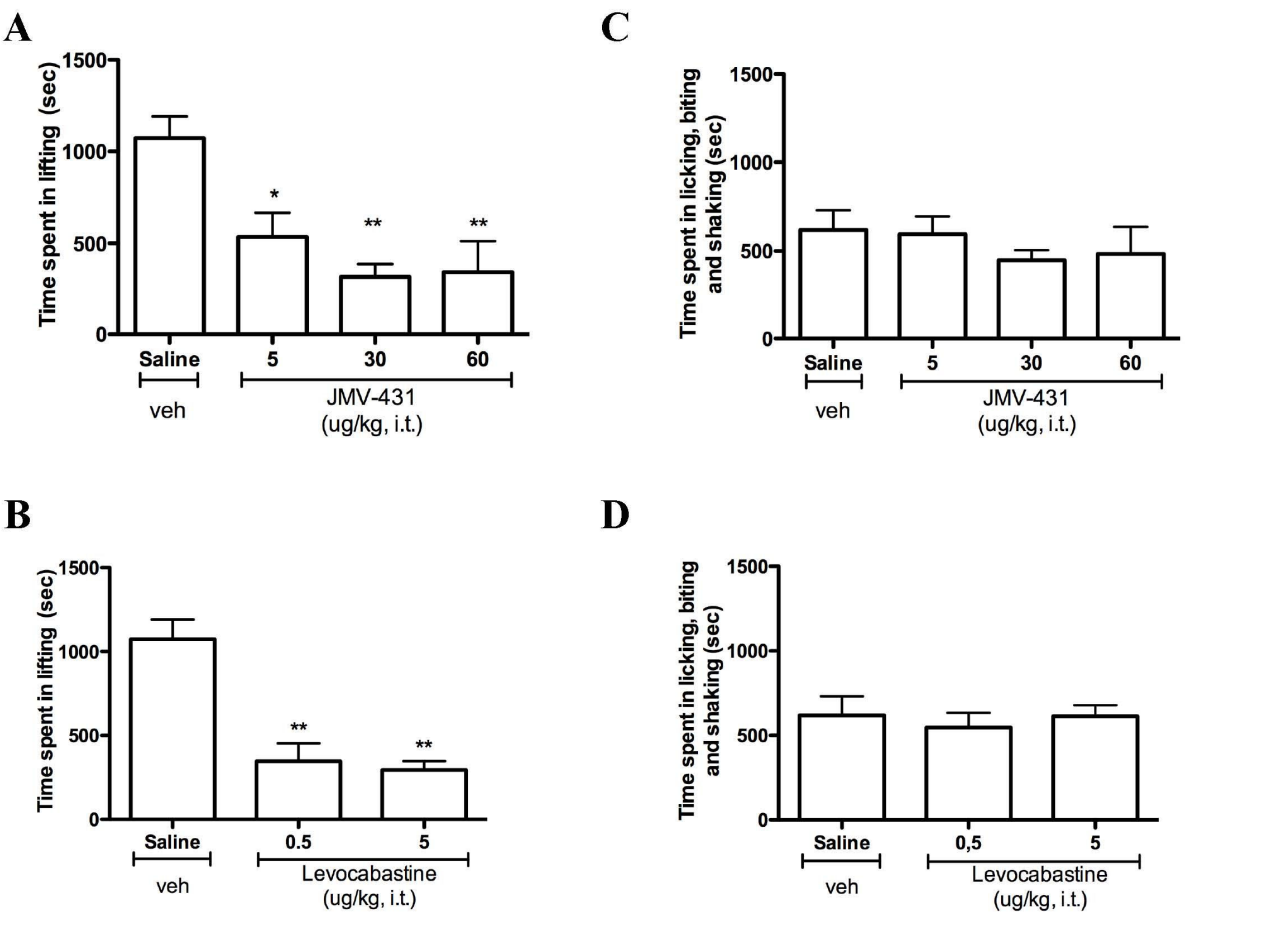
**Effects of NTS2 selective agonists on stereotypic behavior reactions to formalin**. The cumulative nociceptive response time of lifting, licking, shaking and biting the injected paw was measured during the second phase (21–60 min). Both NTS2 agonists JMV-431 **(a, b) **and levocabastine **(c, d) **significantly reduce phase 2 lifting but do not reverse formalin-evoked licking/shaking/biting behaviors. The vertical bars denote SEM. * *P *< 0.05 and ** *P *< 0.01 when compared with the saline-treated group (ANOVA followed by Dunnett's multiple comparison test).

Pain-related behaviors elicited by formalin injection may also be quantified by monitoring the duration of episodes of different behavioral endpoints [46]. Such lifting, flinching, licking or biting specific behavioral responses are integrated at different central nervous system levels upon persistent nociception, involving the activity of spinal and/or supraspinal nociceptive circuits [50-52]. By focusing on the inflammatory phase, we then evaluated the effects of i.t. pretreatment with NT agonists on the stereotypic behavior reactions to formalin (Fig. 3). At all doses tested, both selective NTS2 agonists JMV-431 and levocabastine markedly decreased the cumulative time spent in lifting, reaching 71 ± 7% and 73 ± 5% of inhibition, respectively (*P *< 0.05–0.01; Figs. 3A, B). In contrast to the effects on lifting, none of the doses of either JMV-431 or levocabastine had a significant effect on formalin-induced flinching, licking and biting responses, pointing out the role of NTS2 in spinal modulation of persistent nociception (Figs. 3C, D).

**Figure 4:**
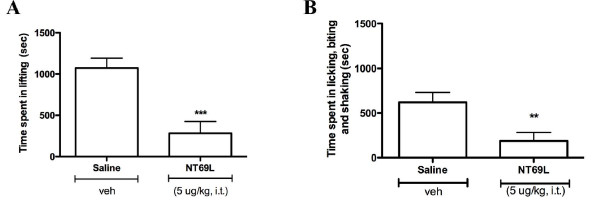
**Antinociceptive response to NT69L in the formalin tonic pain model**. Intrathecal administration of the non-discriminative NTS1-NTS2 agonist, NT69L significantly blocks phase 2 lifting **(a) **but also abolishes the other specific behavioral **(b) **observed during the inflammatory phase. Values represent means ± SEM. ** *P *< 0.01 and *** *P *< 0.001 when compared with the vehicle-treated group (ANOVA followed by Dunnett's multiple comparison test).

Interestingly, the non-discriminative agonist NT69L had a different action profile in reversing the spontaneous aversive behaviors (Fig. 4). Thus, NT69L significantly reduced by 74 ± 13% the lifting time elicited by formalin during the tonic phase (*P *< 0.001; Fig. 4A). However, this peptide was also effective in attenuating the flinching, licking and biting responses (70 ± 15% maximum antagonism, *P *< 0.01; Fig. 4B), indicating that this NT analog affects both spinally and supraspinally processed pain-related behaviors.

### Effects of intrathecal pretreatment with NTS2 agonists on formalin-evoked c-fos-like immunoreactivity in spinal cord

**Figure 5:**
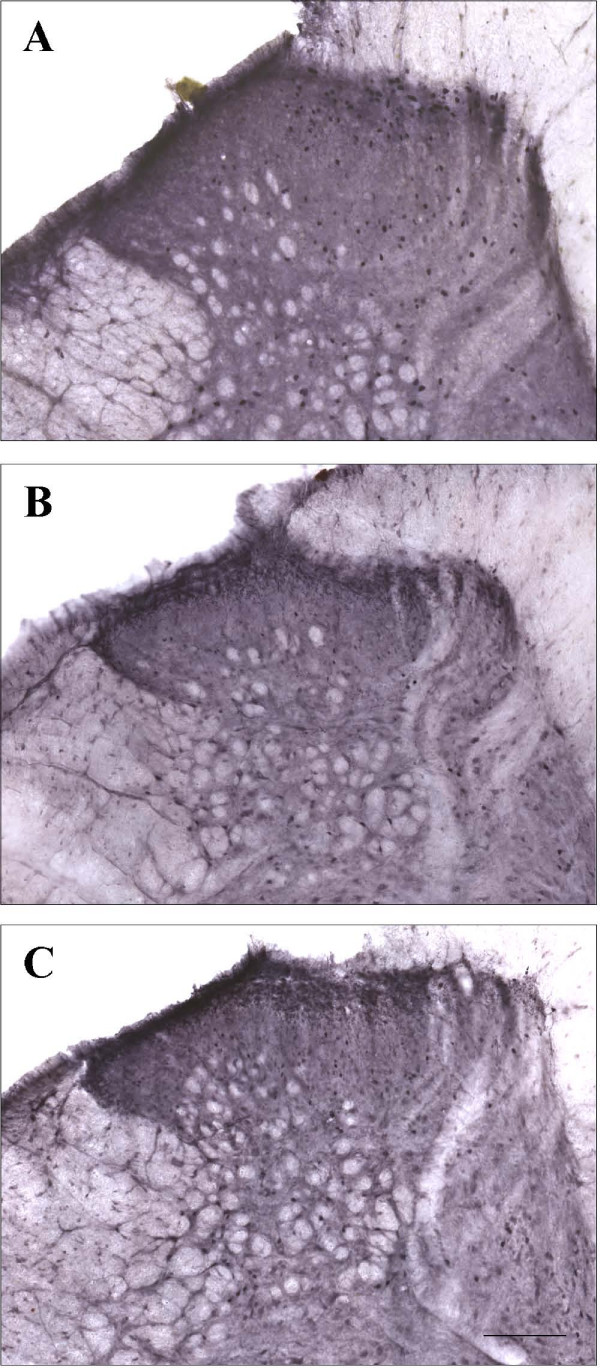
**C-*fos*-like immunoreactivity at the L4-L5 spinal segment ipsilateral to the hindpaw injection of formalin in ipsilateral lumbar sections of the dorsal horn of the spinal cord**. Three different situations are represented (all animals were injected with formalin): control rats that received saline **(a) **and rat receiving either JMV-431 **(b) **or levocabastine **(c)**. Note that both JMV-431 and levocabastine reduced the level of formalin-evoked c-*fos *expression in the superficial dorsal horn. Scale bar: 200 μm.

**Figure 6:**
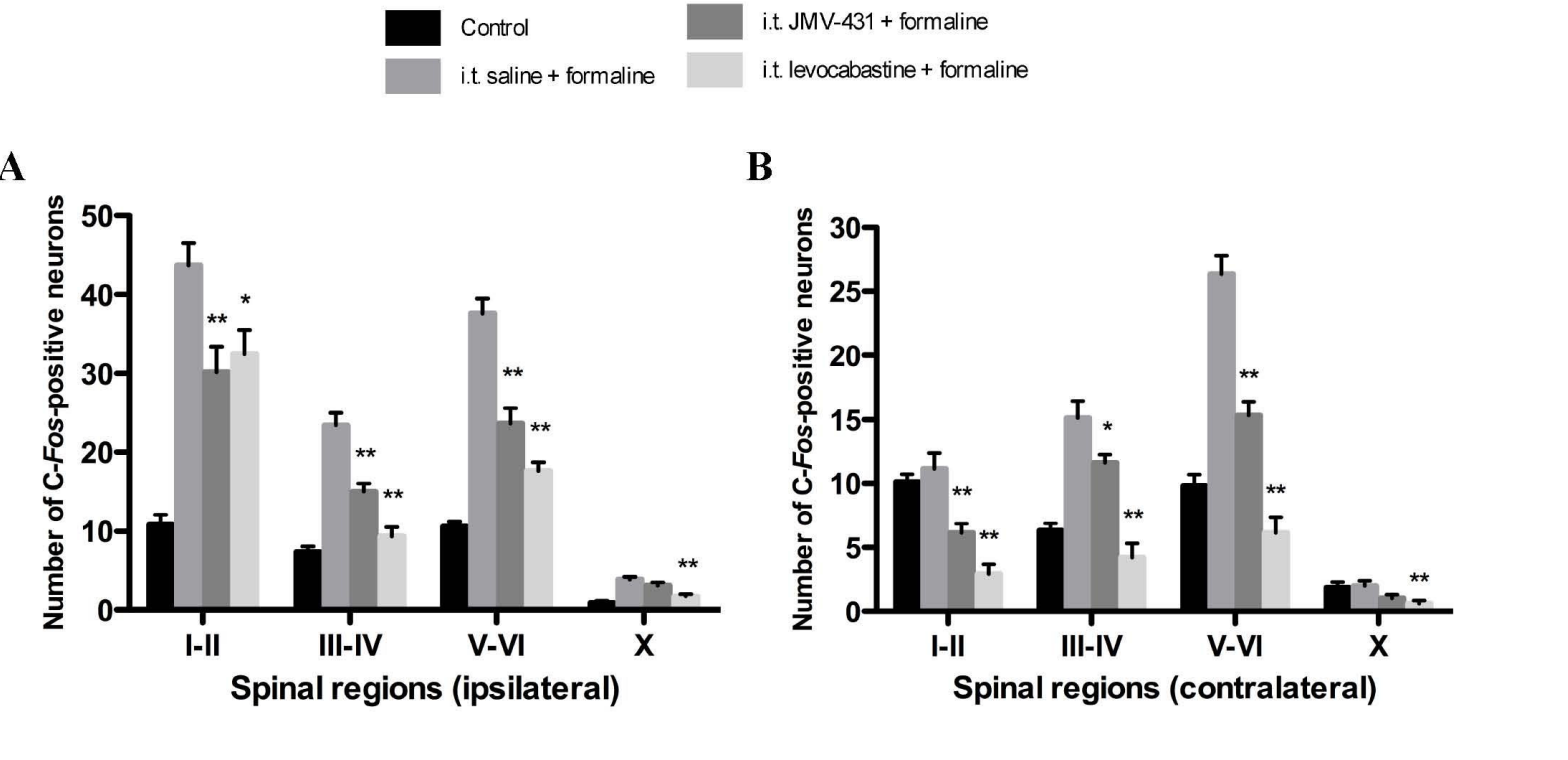
**Regional analysis of the effects of NTS2 agonist administration on formalin-induced c-*fos *expression in spinal cord**. Data represent the mean ± SEM of c-*fos*-positive neurons in the superficial laminae (laminae I-II), nucleus proprius (laminae III-IV), neck of the dorsal horn (laminae V-VI), and lamina X of the L4-L5 segments, both ipsilateral **(a) **and contralateral **(b) **to formalin injection. Formalin increased c-*fos *expression in rats pretreated with saline compared to that for naïve rats. JMV-431 (30 μg/kg) and levocabastine (5 μg/kg) reduced the number of c-*fos *positive neurons in laminae I-II, III-IV and V-VI on the ipsilateral side. Both NTS2 agonists also reversed the spinal neuronal activity observed contralaterally. Statistical analysis between saline and agonist-treated groups: * *P *< 0.05 and ** *P *< 0.01; ANOVA followed by Dunnett's multiple comparison test.

The expression of the immediate early gene c-*fos *and of c-*fos *protein in the spinal cord dorsal horn constitutes a marker of neuronal activity that can be induced by noxious stimuli [53]. Thus, c-*fos *immunohistochemistry was performed on lumbar spinal dorsal horn sections to characterize at the cellular level the relationship between the pain-relieving effect of NTS2 agonists and the biochemical changes of spinal nociceptive transmission (Figs. 5, 6). As previously documented [47], tonic noxious chemical stimulus produced by unilateral subcutaneous formalin injection evokes c-*fos *expression in spinal cord neurons (Fig. 5A). The pattern of nuclear c-*fos*-like immunoreactivity (c-*fos*-LI) was consistent with the known nociceptive primary afferent input from the hindpaw. Indeed, dense labeling was detected, at the L4-L5 segmental levels, principally in neurons located in the superficial dorsal horn (laminae I and IIo) and in the neck of the dorsal horn (laminae V and VI). A scattered staining was also recorded in the nucleus proprius (laminae III and IV), a region that predominantly contains cells only responsive to innocuous stimulation. As described previously [37,54], only few labeled neurons were present in both sides of the spinal cord of non-stimulated rats (i.e. without formalin injection; Fig. 6).

We then evaluated the ability of JMV-431 and levocabastine to suppress formalin-induced c-*fos *protein expression. Spinal administration of JMV-431 markedly decreased formalin-evoked c-*fos*-LI in all laminae (Fig 5B). At a dose of 30 μg/kg, injection of JMV-431 reduced the number of c-*fos*-LI neurons in the ipsilateral laminae I-II, laminae III-IV and laminae V-VI by 31 ± 7%, 36 ± 5% and 37 ± 5%, respectively (*P *< 0.001 compared with control group; i.e rats receiving i.t. saline and formalin injection; n = 4) (Fig 6A). Accordingly, treatment with the other NTS2 agonist, levocabastine also reversed the activation of medullary neurons after the formalin-induced inflammation (Fig. 5C). Quantitative analysis revealed that c-*fos*-LI neurons in ipsilateral laminae I-II, laminae III-IV, laminae V-VI and laminae X were reduced by 26 ± 7%, 60 ± 5%, 53 ± 3% and 56 ± 8%, respectively, following administration of levocabastine (5 μg/kg; *P *< 0.05 – 0.001 compared with control group; n = 4) (Fig 6A). The non-selective agonist NT69L also attenuated the spinal nociceptive neuronal responses induced by formalin injection (not shown). The number of c-*fos*-LI neurons evoked by formalin in the superficial layers, the nucleus proprius, and the neck of the dorsal horn of the ispilateral side was greater than that in the contralateral area. Both NTS2 agonists were, however, efficient to bilaterally counteract formalin-induced neuronal activation (Fig. 6B). The contralateral immunoreactivity of c-*fos *was reduced by pre-treatment with either JMV-431 (laminae I-II (45 ± 7%), laminae III-IV (23 ± 4%) and laminae V-VI (42 ± 4%); *P *< 0.05 – 0.001 *versus *control group; n = 3) or levocabastine (74 ± 7%, 72 ± 7%, 77 ± 5% and 98 ± 1% of reduction in laminae I-II, laminae III-IV, laminae V-VI and lamina X, respectively; *P *< 0.001 *versus *control group; n = 3).

## Discussion and conclusions

The majority of studies on pain processing have used, as a behavioral model of pain, the withdrawal reflex threshold of a limb provoked by a brief noxious stimulus, usually thermal, mechanical, or electrical. Although such traditional nociceptive tests have considerably improved our knowledge of pain mechanisms, the relevance of these models for understanding activity in chronic pain states is somehow limited. Indeed, pain associated with injury and disease is long lasting and frequently associated with inflammation and psychological distress. The molecular and cellular changes observed in nociceptive modulatory circuits provide evidence of this complex functional reorganization occurring in the presence of persistent pain [55,56]. Furthermore, there is a clear indication that suppression of phasic and tonic pain can involve distinct mechanisms [57,58]. In this regard, the formalin test was developed as a model of tonic pain that has a greater relevance to clinical pain than an acute phasic pain test [42,46]. The local injection of formalin into the hindpaw produces a characteristic biphasic behavioral response [44,59]. The first phase of activity reflects a direct activation of sensory afferents and may thus be of limited value in understanding mechanisms involved in persistent pain. A particular focus of the present study was therefore on the second phase, which corresponds to ongoing peripheral activity and involves central sensitization [42,43].

We have recently implicated NTS1 receptors in NT's analgesic effects in tonic spinal pain paradigms [37]. However, the experiments performed with the selective NTS1 antagonist SR48692 have suggested that another NT receptor may participate to the regulation of pain signaling, SR48692 partly reversing the analgesic effects of the non-discriminative agonist NT69L. To our knowledge, the present investigation demonstrates for the first time that NTS2 agonists given intrathecally produce antinociception in the formalin model of persistent inflammatory pain (Fig. 1). These results are in agreement with previous studies suggesting that NTS2 receptors play a role in the regulation of spinal nociceptive inputs. Thus, administration of the selective NTS2 agonists, JMV-431 and levocabastine, into the subarachnoid space of the lumbar spinal region elicited analgesia in the tail-flick test [15,23]. The dose effect seen here with levocabastine was previous reported in different acute and tonic pain paradigms [15,18]. The association of NTS2 receptors with different sub-populations of DRG neurons and their presence within the dorsal horn of the spinal cord also reinforced the involvement of NTS2 receptors in the control of incoming nociceptive messages. Indeed, NTS2 receptors are expressed by substance P- and lectin B4-containing small ganglion cells as well as by large-sized NF200-positive neurons documented to carry primary nociceptive sensory modalities and proprioceptive allodynia, respectively [15]. A dense plexus of NTS2-immunoreactive neuronal processes and numerous NTS2-expressing nerve cell bodies were also detected in the superficial laminae of the dorsal horn and in deeper layers III-V, suggesting that NTS2 may play an important role in modulating the activity of spinal neurons [15].

Since the introduction of the formalin test, different approaches have been used to assess formalin-induced pain-related behaviors [42,50]. The initial behavioral scoring in rats involved assigning of weighted scores to different kind of behaviors, the intensity of each nociceptive-related activity being directly correlated in proportion to their category weights [43,44]. Based on the concept that the different behavioral endpoints represent a one-dimensional view of nociception, this method reflects the overall pain experience of the animal being tested. Using the weighted average pain scores, we found that NTS2-selective agonists and non-discriminative compounds gave rise to different action profiles during the inflammatory phase of the formalin test. Indeed, both JMV-431 and levocabastine had higher nociceptive scores than did NT69L (which binds to both NTS1 and NTS2) during the early phase 2 (21–39 min), while all drugs were efficient in reducing pain during the late phase 2 (40–60 min) (Fig. 2). Accordingly, we recently demonstrated that the selective-NTS1 agonist, PD149163 behaved as NT69L, suppressing pain-related behavioral activity in both early and late parts of phase 2 [37]. The decrease in the analgesic efficacy of NTS2-selective agonists observed during the first half of phase 2 may be related to the non-involvement of NTS2 during the early tonic inflammatory responses. This hypothesis is supported by previous findings showing that some receptors involved in the regulation of pain processing differently modulate the early and late tonic phases 2 [45,60]. For example, the synthetic opioid analog, loperamide, only abolishes flinching behaviors in the second part of phase 2 [60]. Alternatively, it may also reflect the active participation of NTS2 receptors during the interphase. We could indeed speculate that the endogenous release of NT acting on NTS2 sites during the interphase may reduce or mask the analgesic effects of NTS2 agonists at the beginning of the inflammatory phase. This delay in the antinociceptive effects of NTS2-selective agonists possibly corresponds to the time frame required for functional resensitization of NTS2 receptors. This second hypothesis is reinforced by previous studies showing that the interphase of the formalin test is the result of active endogenous pain-inhibitory mechanisms [61-67].

Aside from the weighted-scores technique, pain intensity elicited by formalin injection may also be rated by the assessment of each individual nociceptive behavior, such as the total time the paw is kept elevated from the floor [68,69], the number of times the rat flinches in a given time period [57,59], or the time spent licking and biting the injected paw [70]. A problem which may occur when measuring only one behavior such as lifting is that if a given treatment prevents this behavior, it is considered to produce 100% analgesia, despite the possibility that the rat may still exhibit other characteristic nociceptive behaviors, such as licking and biting [43]. However, the advantage of single parameter recordings over the weighted-scores method is that by taking into account the measurement of multiple behaviors, it would be possible to assess nociceptive behaviors in a manner that reflects the multidimensional nature of pain experience [42,46,50,59]. Based on this method of analysis, we demonstrated that NTS2-selective agonists behaved differently over the inflammatory phase than non-discriminative compounds and could therefore influence pain transmission in a distinctive manner. Thus, spinal delivery of JMV-431 and levocabastine significantly attenuated formalin-induced lifting but not licking, biting and shaking responses during the inflammatory phase (Fig. 3). Unlike NTS2 drugs, the NT69L agonist, acting on both NTS1 and NTS2 receptors, was able to reverse all nociceptive endpoint behaviors observed following tissue injury by intraplantar formalin (Fig. 4). In Sprague-Dawley rats, these stereotypic behavioral reactions to formalin have been used extensively to evaluate analgesic properties of drugs, and different effects on lifting, shaking, licking, and biting have also been observed with other agents [46,50]. Systemic naloxone was indeed shown to increase formalin-induced flinching while simultaneously decreasing licking behavior [71]. Furthermore, peripheral injection of NMDA receptor antagonists significantly attenuated formalin-induced lifting, while flinching behavior was not affected [72]. This diversity of outcomes observed in the formalin test was also reported with other spinally administered analgesics and antidepressants [73,74]. Even so, concerns regarding motor effects influencing formalin behaviors have been expressed. It has been advocated that licking/biting behaviors are subjected to motor influences and stereotypy, whereas flinching (e.g. paw elevations and paw shakes) is a more spontaneous response, being less influenced by other non-nociceptive behavioral changes (e.g. motor) [42,46,59]. In this study, we focused on doses that produced no observable motor impairment [75]. Consequently, it is unlikely that the suppression of licking/biting behaviors induced by medullary delivery of non-selective NTS1/NTS2 compounds is related to alterations in motor performance.

The differential effects of pure NTS2 agonists in eliciting lifting and biting/licking responses noted here also suggest that theses behaviors involve distinct mechanisms or neural circuitry. Accordingly, pain-related behaviors can be associated with distinct brain structures, including spinal, brainstem, and cerebrally mediated responses to nociceptive stimulation [76]. Well-known for evaluating the effects of analgesic treatments on long-lasting pain, the formalin test also allows the recording of behavioral reactions relayed at different levels of the central nervous system [50]. Aside from the spinal cord, formalin-evoked responses seem to be mainly integrated in the brainstem and midbrain, as decerebration and decortication do not affect a range of behaviors [77,78]. Regarding the stereotypical nocifensive behaviors observed following formalin administration, it was shown that the persistence of limb flexion is largely a spinal reflex, as it can be evoked following chronic spinalization [79]. Conversely, supraspinal influences have been proposed to contribute in licking/biting behavioral manifestations associated with pain [74,80,81]. With this perspective, we could therefore hypothesize that NTS2 agonists, reducing spontaneous pain manifested by paw lifting, may specifically recruit spinal endogenous antinociceptive systems. Alternatively, the cumulative effects of NT69L (which binds to both NTS1 and NTS2) on lifting and licking/biting behaviors may reveal that NTS1 receptor activation modulates supraspinal nociceptive networks following formalin-induced tissue injury.

Persistent nociceptive stimulation of primary sensory afferents causes prolonged alterations in the neurochemistry and phenotype of postsynaptic dorsal horn neurons. It enhances their excitability leading to hyperalgesia and allodynia that develop after tissue injury. Accordingly, noxious stimuli initiate specific spatio-temporal patterns of *c-fos *expression within the spinal cord, allowing mapping of spinal nociresponsive neuronal populations (including projection cells and both excitatory and inhibitory interneurons) [53,54]. In the present study, we used *c-fos *expression as an anatomical correlate of the behavioral measures to evaluate the antinociceptive activity of NTS2 compounds. Our results revealed that intrathecal NTS2 agonists reverse formalin-induced activation of c-*fos*-like immunoreactivity within the dorsal horn (Figs. 5, 6). Specifically, we found that JMV-431 and levocabastine significantly reduced c-*fos*-LI within the superficial layers of the rat spinal cord (Rexed's laminae I-II), and also exerted inhibitory effects on noxiously evoked *c-fos *expression in deeper laminae. In view of its spinal distribution [15], NTS2 receptor-mediated inhibition of nociceptive transmission may occur at multiple laminae levels.

Converging evidence supports the idea that noxious inputs to the spinal cord are actively modulated by descending systems that serve not only to inhibit, but also to facilitate nociception [52]. The participation of these suprapinal sites is of great clinical significance as it is now apparent that the development and maintenance of exaggerated pain states may result from an alteration of the balance between facilitatory and inhibitory brain circuits [82]. During the formalin response, the descending pain-control influences originating from the rostroventromedial medulla are indeed essential in maintaining the nociceptive behavioral manifestations [52]. The mechanisms by which NT receptor agonists induce analgesia may therefore arise from local spinal pain inhibition or regulation of supraspinal inputs. As spinal activation of NTS2 receptors was shown here to block specifically the nociceptive behaviors integrated at the spinal cord level, we might propose that spinal delivery of NTS2 agonists interrupts conduction of the nociceptive processing by reducing excitability of peripheral primary afferent terminals and inhibits dorsal horn c-*fos *nociresponsive interneuron activity. However, since non-discriminative agonists reversed supraspinal nocifensive behaviors, the antinociception observed could also rely on the inhibition of the spino-bulbo-spinal loop by NTS1 receptor activation. It is currently thought that this inhibitory drive is due to a reduction in the activity levels of c-*fos*-positive neurons projecting to upper brainstem structures [37].

To our knowledge, the present study is the first to demonstrate that activation of NTS2 receptors induces analgesia in a persistent pain model. The intrathecal administration of NTS2 drugs was shown to reduce behavioral and molecular markers of persistent inflammatory pain. Our results also suggest that a dichotomy exists between the spinal antinociceptive actions of NTS2-selective agonists and NTS1/NTS2 mixed compounds. Further research to design NTS1 and NTS2-selective novel analogs that cross the blood-brain barrier may therefore offer new avenues for the treatment of chronic pain.

## Competing interests

The authors declare that they have no competing interests.

## Authors' contributions

GR carried out the behavioural and immunohistological experiments, data analysis and drafted the manuscript. MAD participated in the behavioral experiments. SB participated in immunohistological experiments. FE carried out binding experiments. JM and ER developed neurotensinergic analogs. KB carried out technical assistance. NB participated in the coordination of the study and drafted the manuscript. PS conceived the study and participated in its design, coordination and wrote the final version of the manuscript. All authors have read and approved the final manuscript.
